# Fecal sacs do not increase nest predation in a ground nester

**DOI:** 10.1007/s10336-018-1566-8

**Published:** 2018-05-29

**Authors:** Enrique Rubio, Olivia Sanllorente, B. Irene Tieleman, Juan Diego Ibáñez-Álamo

**Affiliations:** Groningen Institute for Evolutionary Life Sciences, University of Groningen, 9700 CC Groningen, The Netherlands

**Keywords:** Nest sanitation, Visual cues, Nest predation hypothesis, *Lullula arborea*, Nest concealment, Woodlark

## Abstract

Most altricial birds remove their nestlings’ feces from the nest, but the evolutionary forces driving this behavior are poorly understood. A possible adaptive explanation for this could be that birds avoid the attraction of nest predators to their nests due to the visual or olfactory cues produced by feces (nest predation hypothesis). This hypothesis has received contrasting support indicating that additional experimental studies are needed, particularly with respect to the visual component of fecal sacs. To test this hypothesis, we conducted an experiment manipulating the presence of fecal sacs on inactive Woodlark (*Lullula arborea*) nests. This ground nester has highly cryptic nests that are mainly depredated by visually oriented nest predators (i.e., corvids) in our study population, making it an excellent system to test for the nest predation hypothesis. Our results showed that the presence of fecal sacs in the nest does not seem to be an important factor explaining nest predation. Interestingly, the effect of nest concealment, the most important factor explaining nest predation in Woodlark nests, depended on whether the nest was depredated the previous year or not, supporting the importance of using different nesting sites between years. Our findings indicate that this important nest sanitation behavior is not likely motivated by nest predation and highlight the need to explore alternative selective pressures in this context.

## Zusammenfassung

**Kotballen führen bei Bodenbrütern nicht zu mehr Nestraub.**

Bei den meisten Nesthocker-Arten entfernen die Altvögel die Ausscheidungen ihrer Jungen aus dem Nest, aber die evolutionären Kräfte hinter diesem Verhalten sind noch weitgehend unbekannt. Eine mögliche Erklärung des Anpassungsvorteils dieser Verhaltensweise könnte sein, dass die Vögel von den Ausscheidungen ausgehende optische oder olfaktorische Reize, die die Aufmerksamkeit von Nesträubern auf ihr Nest lenken könnten, beseitigen (Nesträuber-Hypothese). Diese Hypothese findet allerdings nur gemischte Unterstützung, was zeigt, dass hier weitere experimentelle Untersuchungen vonnöten sind, vor allem hinsichtlich des optischen Aspekts der Kotballen. Um diese Hypothese zu prüfen, führten wir ein Experiment durch, in dem wir bei inaktiven Nestern der Heidelerche (*Lullula arborea*) das Vorhandensein von Kotballen manipulierten. Diese Bodenbrüter bauen sehr versteckte Nester, die in unserer Versuchspopulation überwiegend von sich optisch orientierenden Nesträubern (z. B. Rabenvögeln) geplündert wurden, und somit ein sehr gutes Test-System für die Nesträuber-Hypothese darstellten. Unsere Ergebnisse zeigten, dass das Vorhandensein von Kotballen im Nest kein wichtiger Faktor bei der Nesträuberei ist. Interessanterweise hing die Bedeutung, wie gut das Nest versteckt ist, der Nestraub am besten erklärende Faktor, davon ab, ob das Nest im Vorjahr ausgeraubt wurde oder nicht. Dies unterstreicht, wie wichtig es ist, in unterschiedlichen Jahren unterschiedliche Nistplätze zu nutzen. Unsere Ergebnisse weisen darauf hin, dass dieses wichtige Nestsäuberungsverhalten nicht unbedingt mit Nesträuberei erklärt werden kann, und unterstreichen die Notwendigkeit, nach anderen Selektionsmechanismen dafür zu suchen.

## Introduction

Nest sanitation is an important component of parental care, widely present in birds but still poorly understood (Ibáñez-Álamo et al. 2017). The removal of nestling excrements, probably one of the main nest sanitation activities carried out by altricial birds (Guigueno and Sealy 2012), has received increasing attention in the last years with a special focus on experimental studies exploring the adaptive significance of such behavior (e.g., Ibáñez-Álamo et al. 2013, 2014a; Quan et al. 2015). In most of the species, the nestlings’ feces are encapsulated in a mucous covering (the fecal sac) (e.g., Herrick 1900; Pycraft 1909; Thompson 1934) which facilitates their manipulation by parents (Ibáñez-Álamo et al. 2017). Furthermore, the removal of the nestlings’ feces has been suggested to drive the evolution of fecal sacs (Ibáñez-Álamo et al. 2017), which emphasizes the importance of nest sanitation for life history traits in altricial birds.

One of the main hypotheses proposed to explain the removal of nestling feces from the nests of altricial birds is the nest predation hypothesis, which states that the presence of feces in the nest will attract predators (Herrick 1900; Weatherhead 1984; Petit and Petit 1987). Nest predation is a key factor modulating parental care behaviors in birds such as incubation or food delivery to nestlings (e.g., Ghalambor et al. 2013; Morosinotto et al. 2013; Hua et al. 2014; reviewed in Martin and Briskie 2009; Ibáñez-Álamo et al. 2015). According to this hypothesis, the presence of feces in active nests could attract nest predators due to visual or olfactory cues (Ibáñez-Álamo et al. 2014a). Visual cues would be due to the white part of the fecal sac and would likely be easily detected by visually oriented predators (i.e., birds), whereas the olfactory cues would likely favor the attraction of olfactory oriented predators (i.e., mammals). However, several studies showed that different visual cues in the nest do not increase nest predation significantly, as visual predators tend to detect nests rather than their contents (Götmark 1992; Weidinger 2001). To our knowledge, there are only two published experiments exploring the nest predation hypothesis and they provide contrasting results. In the first experimental study on nest sanitation, Petit et al. (1989) demonstrated that the presence of feces close to artificial nests increased their predation. However, the authors suggested that their results were difficult to interpret because of the artificial nature of their experiment, which involved using non-specific artificial ground nests and chicken feces covered with a mixture of water and flour. More recently, another experimental study using real nestling feces and active Common Blackbird (*Turdus merula*) nests, found no support for the attraction of nest predators due to feces (Ibáñez-Álamo et al. 2014b). This study, however, tested the olfactory component of the feces exclusively. Therefore, additional studies also considering the visual component of nestling feces are required to test whether fecal sacs really attract nest predators.

In order to test the nest predation hypothesis, we experimentally manipulated the presence of feces in inactive Woodlark (*Lullula arborea*) nests. This is an ideal species in which to test the nest predation hypothesis, and particularly the effect of visual cues of fecal sacs, as it suffers from an elevated nest predation pressure by visually oriented predators (Praus et al. 2014) and has evolved several adaptations to avoid it, including highly cryptic nests (Donald 2017). In addition, adult Woodlarks remove all their nestling feces (Blair and Tucker 1941), indicating that their presence in the nest might be a risk factor potentially increasing their nest predation risk. We predicted that nests with fecal sacs would be significantly more preyed upon than those without them.

## Methods

The study was conducted in Aekingerzand, within the Drents-Friese World National Park, in the north of the Netherlands (52°56′N, 6°17′E) during April–June of 2016. This is a large area of heather, grass, moss, and bushes surrounded by coniferous forest where Woodlarks breed from March to July (Hegemann 2012). The main predators of Woodlark nests in the study site are visually oriented corvids (Carrion Crows *Corvus corone* and Eurasian Jays *Garrulus glandarius*) (Praus et al. 2014).

We searched for active Woodlark nests in our study area from the beginning of the breeding season. Adults carrying nest material or food in the beak were found by direct observation and followed to the nest. All nests found were visited regularly (every 3 days) until hatching. Once a nest was depredated during the incubation stage, it was collected and stored in a plastic bag in the field station until its utilization. A nest was considered as preyed upon when no egg remains were left in the nest. We only collected nests depredated during the incubation stage (and not during the nestling stage) to avoid potential confounding effects of having some nests with the scent of nestlings and others without it. Using this procedure, we collected 60 depredated Woodlark nests in total.

We placed these depredated nests on known Woodlark nesting locations from the previous year (2015) that had been marked with a Global Positioning System device, therefore using real sites selected by Woodlarks. Information on whether nests were preyed upon or successful in these locations the previous year was also available. All inactive nests were baited with two Japanese Quail eggs (*Coturnix japonica*) because they are also cryptic and laid in cryptic nests on the ground, therefore minimizing the influence of additional visual cues in our experimental setup. Furthermore, two Japanese Quail eggs (mean volume = 49.9 cm^3^) also offered a similar energetic reward for nest predators as an entire Woodlark clutch of four eggs [mean volume = 37.2 cm^3^; mean clutch size in the population 4.02 eggs (Horrocks et al. 2014)]. Once the nest was placed on its location, we performed the following treatments following a similar experimental design as that used by Ibáñez-Álamo et al. (2014a). The first treatment comprised an experimental group to which we added two fecal sacs at the rim of the nest at every visit in order to mimic the natural accumulation of feces in an unattended nest. In the second treatment, comprising the manipulation control group, we added a similar weight of mud (mean ± SE 2.37 ± 0.26 g; obtained from the vicinity of the nest) as the excrements added to the experimental group previously described (mean ± SE 2.38 ± 0.18 g; linear model, *F* = 0.0002; *df* = 1; *p* = 0.99). The main visual difference between feces and mud was the conspicuous white part typical of fecal sacs. The third treatment comprised a control group that was visited in a similar way but to which nothing was added.

Blackbird fecal sacs were used for the experimental nests due to the low availability of Woodlark nests with chicks from which to collect Woodlark feces. Blackbird fecal sacs do not attract predators to nests due to their olfactory cues (Ibáñez-Álamo et al. 2014a) and offer similar visual cues as Woodlark fecal sacs (personal observation). Blackbird nestlings easily defecate when handled (Ibáñez-Álamo et al. 2014b). Once collected, fecal sacs were preserved cold (4 °C) in a small container with water and added to the nests within the following 24 h. This method allowed us to mimic freshly produced fecal sacs keeping intact their mucous covering and water content. This is particularly important as alterations in these factors could potentially affect their detectability. All nests were visited every 2 days during a 9-day period or until they were depredated. This 9-day period is the mean duration of the nestling period for Woodlarks in our population (Praus et al. 2014). We considered a nest depredated if the eggs were either broken or missing. Finally, as nest predation may vary during the breeding season (Picman and Schriml 1994; Weidinger 2001), to avoid a temporal bias in our findings we distributed our inactive nests in four temporal groups (starting 25 April, 4 May, 20 May and 15 June). Each temporal group consisted of 15 nests, five per treatment.

We also calculated a visibility index for each nest using a categorical variant of a method previously published (Bayne and Hobson 1997). The same observer (E. R.) graded (0–2) the visibility of each nest to the human eye from a distance of 2 m in each of the four cardinal directions. The sum of the values obtained in each cardinal point established the visibility index of the nest (range 1–6 in our dataset). The visual score was calculated as: 0, when neither the nest nor the eggs could be seen; 1, when part of the nest or the eggs could be seen but not completely; 2, when the nest and the eggs could be seen completely.

We tested the effect of our treatment on the daily survival rate of our inactive Woodlark nests by using a model selection based on the second-order Akaike information criterion (AICc) (Burnham and Anderson 2002) and the packages R Mark (version 2.2.4; Laake 2013) and lubridate (version 1.7.1.; Grolemund and Wickham 2011). R Mark is an interface to run nest survival models in the program MARK (White and Burnham 1999). As additional predictors, we also included in the model selection the visibility index, the previous-year status [depredated (1) or not (0)], temporal trial (1–4) and all two-way interactions. We used the package MuMin (version 1.40; Bartoń 2017) to calculate the model-averaged coefficients and relative importance of each predictor of those models with a weight > 1%. The analyses were done using R software (version 3.4.2; R Core Team 2017).

## Results

Our results indicate that the best model explaining the effect of the presence of fecal sacs is that containing the interaction of previous-year status by visibility index (Table 1). The model that includes the same interaction in addition to the temporal trial, as well as that with the latter as the only predictor are also considered equally parsimonious (ΔAICc <2) (Burnham and Anderson 2002) though they have a smaller weight (Table 1). The interaction of previous-year status by visibility index is also among the most important predictors, in addition to the other individual variables included in the most parsimonious models (Table 2). The model-averaged coefficients indicated that the increased predation associated with a low nest concealment (high visibility index) only applies to those sites whose Woodlark nests were depredated the previous year. On the contrary, we found very little support that our experimental treatment on nest predation affected the daily survival rate of the inactive Woodlark nests (Tables 1, 2), suggesting that fecal sacs did not increase the probability of nest predation (Table 3).

**Table 1:** Model selection results from the R Mark analysis of daily survival rate indicating the number of parameters considered in the model (*K*), Akaike information criteria value corrected for small sample sizes (*AICc*), the difference in AICc values between a given model and that with the lowest AICc value (Δ*AICc*), and the Akaike weights for each model

Model	*K*	AICc	ΔAICc	Weight
Previous year status × visibility	4	162.05	0.0	0.24
Previous year status × visibility + temporal trial	5	162.27	0.21	0.21
Temporal trial	2	163.81	1.76	0.10
Visibility	2	164.22	2.16	0.08
Constant DSR	1	164.58	2.53	0.07
Previous year status + visibility	3	164.59	2.54	0.07
Previous year status × visibility + temporal trial + treatment	7	164.84	2.78	0.06
Visibility × Temporal trial	4	165.25	3.20	0.05
Previous year status	2	165.63	3.58	0.04
Previous year status × temporal trial	4	166.91	4.85	0.02

Only the ten most supported models are included in the table

*DSR* Daily survival rate

**Table 2:** Model-averaged coefficients (± SE), confidence intervals (*CI*s) and relative importance for each predictor

Predictor	Estimate	SE	2.5% CI	97.5% CI	Importance
Intercept	0.375	1.571	− 2.707	3.457	
Visibility	0.609	0.549	− 0.159	1.768	0.76
Previous year status (1)	1.633	1.697	− 0.584	5.440	0.67
Previous year status (1) × visibility	− 0.494	0.585	− 1.883	− 0.021	0.52
Temporal trial	0.008	0.013	− 0.012	0.046	0.46
Treatment (experimental)	− 0.058	0.236	− 1.523	0.492	0.11
Treatment (manipulation control)	− 0.025	0.183	− 1.217	0.764	
Temporal trial × visibility	− 0.001	0.003	− 0.025	0.011	0.05
Temporal trial × previous-year status (1)	− 0.001	0.004	− 0.055	0.036	0.02

**Table 3:** Daily nest survival (± SE) and overall survival rate (9-day period) for each of the three treatments in our experiment obtained from the minimum model (~ treatment)

Treatment	DSR	SE	Overall survival rate
Experimental	0.894	0.024	0.366
Manipulation control	0.917	0.030	0.459
Control	0.926	0.025	0.500

Sample size for each treatment is 20

## Discussion

Our experiment does not support the nest predation hypothesis and suggests that nestlings’ feces do not attract more predators to the nest, at least in the Woodlark. Our results, therefore, are in agreement with those obtained by another recent experimental study testing this hypothesis (Ibáñez-Álamo et al. 2014a). That investigation, using a similar experimental design did not find evidence to support that the olfactory component of fecal sacs increased the risk of predation of blackbird nests. Here, we found a similar result in a more visually oriented context indicating that the white part of fecal sacs does not seem to promote the detectability of nests. This finding fits with the idea that nest contents are not the key factor used by nest predators in order to look for nests (Götmark 1992; Weidinger 2001). Our results are also in agreement with other observational studies showing that nest predation does not seem to influence other aspects of feces removal like the direction of feces transportation (Weatherhead 1984; Petit and Petit 1987), or additional experimental evidence indicating that it is not an important factor explaining the ingestion of fecal sacs (Ibáñez-Álamo et al. 2013).

On the other hand, our findings contrast markedly with those obtained by Petit et al. (1989) in another experimental study that tested the nest predation hypothesis. The authors of that study already acknowledged that their findings could be an artifact due to the artificial methodology used including unspecific artificial nests (a Quail egg directly placed on the ground) and artificial nestling feces. The use of artificial nests in nest predation studies has been recommended only to test specific hypotheses and once the nest predator community of the focal species has been identified (Ibáñez-Álamo et al. 2015), like in our case, as otherwise they may lead to inaccurate conclusions given the important differences that they may have with natural nests (Major and Kendal 1996; Davidson and Bollinger 2000). Another important difference between Petit et al.’s (1989) study and ours that might explain the opposite results is their use of adult chicken feces mixed with a solution of flour and water in contrast to our use of fresh and natural fecal sacs, which might have attracted in their case an unnatural community of nest predators (i.e., adult birds or chicken predators). In fact, they indicated that even though the visual component of their artificial nestling feces was similar to real fecal sacs, the olfactory component seemed to be completely different. This in addition to the fact that they estimated that the majority of nest predators were (olfactory oriented) mammals led them to suggest that their effect will be attributed to the odor of feces, and therefore, not contradictory to our results. Alternatively, the different results in the two studies could be explained by the effect of the mucous covering of fecal sacs, only present in our study. It might be possible that the mucous covering (not found in adult feces) reduces the detectability of nestling feces by predators, either by reducing their visual or olfactory cues. However, to our knowledge, this possibility has not been investigated so far, and in fact, the adaptive function(s) of this trait is not clear yet (Ibáñez-Álamo et al. 2014b, 2017).

Our results also indicate that the location of the nest might be an important factor determining predation. Some studies on ground nesters suggest that nesting at the same site increases the probability of being preyed upon (Martin et al. 2000; Yahner and Mahan 1996). Initially, our results seem to be contradictory to these latter studies as we found a positive effect of previous-year status on nest survival. However, this effect was mediated by nest concealment in Woodlarks, indicating a dominant role of detectability rather than long-term memory by predators in explaining the between-year repeatability of predation events. Nest concealment has been proposed to have an important effect in other ground nesters too (e.g., Gregg et al. 1994). It is also possible that corvids, which seem to use long-term memory to predate ground nests (Sonerud and Fjeld 1987), can remember more easily less concealed nests between years. In contrast, experiments performed with non-ground nests do not seem to show this between-year consistency with site (e.g., Yahner and Mahan 1999; Weidinger 2001), although there seem to be differences depending on the type of nest predator (Weidinger and Kovcara 2010). These differences between ground and canopy nesters may be due to the different community of predators in these habitats, with different cognitive capacities.

Our study provides an interesting addition to previous knowledge in the field and expands the still low number of experimental studies focused on investigating the adaptive origin of fecal sac removal. We found that nest predation does not seem to be an important selective pressure explaining this relevant nest sanitation behavior in Woodlarks. However, we cannot rule out the nest predation hypothesis completely because of the lower overall survival rate of nests with fecal sacs (Table 3), and the possibility that it applies to other species or systems. Additional studies are clearly required. In order to understand the selective forces driving this widespread avian behavior, future studies should also investigate alternative hypotheses like the antimicrobial hypothesis which states that nestling feces removal would be carried out in order to avoid the negative effects of potentially harmful enteric bacteria (Ibáñez-Álamo et al. 2014b) and the parasitism hypothesis that affirms that the reason for fecal sac removal is because nestling feces attract parasites to the nest (Skutch 1976; Ibáñez-Álamo et al. 2016).
